# India @ 75: Investing in a healthy workforce for a healthy future

**DOI:** 10.1016/j.lansea.2022.100049

**Published:** 2022-08-01

**Authors:** The Lancet Regional Health – Southeast Asia

India has witnessed substantial improvement in the health status of its population during the last 75 years, post-independence. India is considered a pharmaceutical and biotechnological hub, and a promising global health leader in the making; yet it fails to provide adequate care to large parts of the country's growing population, particularly in rural regions. The COVID-19 pandemic has overwhelmed the already stretched Indian health systems, reversing some of the gains made in the past several decades. Besides inadequate infrastructure, the severe shortage of the health workforce is a significant challenge that has become evident during this pandemic. India needs at least 1·8 million doctors, nurses, and midwives to achieve the minimum density of 44·5 health professionals per 10 000 population (currently 21 per 10 000) to provide equitable health care. This gap is further worsened by outmigration of many health professionals. Additionally, most graduating physicians set up private practices in urban areas, exacerbating the disparities between urban–rural and public–private health services.

Many low- and middle-income countries have attempted to implement innovative schemes to attract and retain a rural health workforce, such as recruiting more students from rural backgrounds, increasing the number of medical schools, rural-focused curricula, and fellowships, in addition to ensuring a robust rural health system. The Indian Government is also trying to overcome this shortage by expanding medical college seats and starting special courses for rural health services in addition to launching innovative schemes to attract the health workforce to community service. This strategy alone might bridge the demand–supply gap; however, the bigger challenge is to attract, retain, and motivate the health workforce to enhance the effectiveness and efficiency of the current health systems, that is skewed to the private health sector. Insufficient remuneration, unsafe working environments, and poor governance in health organisations in addition to the absence of reward and recognition, job insecurity, and low priority to health worker needs impacted the retention and work quality of health personnel in India.

Some of the strategies to address health personnel shortages, such as task shifting without adequate training, and multiple deployments, could inadvertently worsen the working conditions of health workers. Studies have documented the elevated risk of stress, burnout, moral injury, depression, trauma, and other mental health challenges among health-care workers in addition to a higher risk of cardiovascular diseases and musculoskeletal disorders. Long shifts, night shifts in succession, and fear of infection seem to increase the risk of sleep disturbances, harmful alcohol use, anxiety, depression, and post-traumatic stress disorder among health workers.

Public health emergencies increase these risks, as evident in emerging research on the negative effects of the COVID-19 pandemic on health-care workers. Women seem to experience more challenges in work–life balance in addition to feelings of guilt and social stigma compared with men. In this issue, we publish a Comment by Asthana and Mayra, detailing the complex struggles of female frontline rural community health workers in India, called ASHAs (hope in Hindi), and make a case for more support for this agency that connects health systems to millions living in rural poverty.

Instances of Indian health professionals on strike due to unsafe working conditions; and suicide among doctors and nurses due to stress, workplace harassment, mental illness, and negative media coverage, have shifted our attention to the much-needed wellbeing of health and care workers. The Indian Government passed an ordinance to protect health workers from violence witnessed during the pandemic, yet this move seems insufficient. Although a welcomed step in the right direction, much more needs to be acted upon to prioritise and protect the health and wellbeing of the Indian health workforce.

Countries, such as Brazil and Thailand, that are undergoing similar economic development as India have implemented several steps to bring about institutional changes to produce more staff, improve their training, enhance working conditions, and strengthen management capacity. The family health team model, under the Sistema Único de Saúde (Unified Health System), in Brazil transformed the orientation of values and practices of multidisciplinary teams of health workers towards primary care. In Thailand, a comprehensive health workforce policy focussing on professional satisfaction to engage health professionals in rural service was developed in 1995. In India, policy makers and government officials need to understand the value of investing in the health and wellbeing of the health workforce, at an individual, organisational, and societal level.

Investing in long-term measures, protecting, and promoting a healthy workforce might involve leveraging existing evidence-based interventions for reducing psychological distress during emergencies; foster a work culture of inclusion, collaboration, and support. Health systems driven by the needs of patients and health-care providers will build a strong foundation for a resilient health system.

According to WHO, a knowledgeable, skilled, and motivated health workforce is critical for achieving universal health coverage. WHO named 2021 as the year of health and care workers to appreciate the sacrifice and service of heroes that served populations in difficult times of COVID-19. When ASHAs were recognised as Global Health Leaders at the 75^th^ World Health Assembly this year, the world saluted the importance of this human workforce that is so crucial in driving key health changes in communities. If India is to move to the next milestone of 100 years with the vision of health for all, it must take much better care of its vast health workforce that will shoulder and drive this vision. Financial support aside, health workers need well-equipped and responsive working environments where they can feel valued and safe, both physically and psychologically. The case to invest today in the Indian health workforce is clear.

